# Prevalence and Risk Factors Associated with* S. haematobium* Egg Excretion during the Dry Season, Six Months following Mass Distribution of Praziquantel (PZQ) in 2017 in the Bafia Health Area, South West Region Cameroon: A Cross-Sectional Study

**DOI:** 10.1155/2019/4397263

**Published:** 2019-07-01

**Authors:** Vicky Daonyle Ndassi, Judith Kuoh Anchang-Kimbi, Irene Ule Ngole Sumbele, Godlove Bunda Wepnje, Helen Kuokuo Kimbi

**Affiliations:** ^1^Department of Zoology and Animal Physiology, Faculty of Science, University of Buea, P.O. Box 63, Buea, SWR, Cameroon; ^2^Department of Medical Laboratory Science, Faculty of Health Sciences, The University of Bamenda, P.O. Box 39, Bambili, NWR, Cameroon

## Abstract

**Background:**

A selective population mass drug administration of PZQ involving school-aged children was carried out in the Bafia Health Area in April 2017. This study investigated the prevalence, intensity, and factors associated with* S. haematobium *egg excretion in this foci during the dry season, six months after the chemotherapy campaign.

**Methods:**

A cross-sectional study including 1001 consenting individuals (aged 3-62 years) was carried out in three localities (Ikata, Bafia, and Munyenge) in the Bafia Health Area between November 2017 and January 2018. Information on sociodemographic, stream usage, and contact behaviour was documented.* Schistosoma haematobium *ova in urine were detected using membrane filtration technique.

**Results:**

The prevalence of* S. haematobium* egg excretion was 8% with a higher level recorded in Munyenge (13.2%) than Ikata (7.5%) and Bafia (2.8%). The difference was significant (p < 0.001). Equally, Munyenge had the highest infection intensity (36.36 range: 2-200) when compared with Ikata (16.25 range: 2-57) and Bafia (8.0 range: 0-8). Although the age group (5–15 years) was significantly (p < 0.001) associated with more exposure to infested water, this group was less likely (OR: 0.42 95% CI: 0.19-0.91) associated with* S. haematobium* egg excretion. The risk of egg excretion increased by 4.79 times (95% CI: 2.20-10.41) and 3.68 times (95% CI: 1.59-8.54) among residents in Munyenge and Ikata, respectively. Similarly, frequency to the stream (> thrice/day) was significantly higher (*χ*^2^ = 58.73; p < 0.001) in Munyenge. Frequent contact (three visits/day) with stream correlated with highest odds of egg excretion (OR: 8.43 95% CI: 3.71-19.13).

**Conclusion:**

The prevalence of* S. haematobium *egg excretion was low during the dry season. This was most likely attributed to the preventive campaign with PZQ and may parallel low transmission potentials in infested waters during this period.

## 1. Introduction

Worldwide, an estimated 218 million people suffer from schistosomiasis, of whom ~90% live in Africa [1]. Two-thirds of these cases are caused by* Schistosoma haematobium*, the etiologic agent of UGS [2]. The potential consequences of* S. haematobium* infection include haematuria, dysuria, nutritional deficiencies, lesions of the urinary bladder, hydronephrosis, stunting (in children) [3], and in adults, infertility, cancer, and increased susceptibility to HIV [2, 4, 5]. Mass drug administration with praziquantel is the mainstay of programs for the control of schistosomiasis morbidity. Targeted control on school children is often advocated; however, this strategy usually excludes adults and pregnant women who are potential reservoirs for UGS in endemic communities [6]. There is a growing recognition that treatment alone is not sufficient for effecting elimination. Additional measures such as reducing exposure to infectious water, moderating availability of intermediate snail host, and decreasing contamination of water with egg-containing urine or faeces will be required to interrupt transmission [7, 8].

A predominant feature of* Schistosoma haematobium* infection is urinary egg excretion. In the current absence of more sensitive field diagnostics, egg counts by microscopic examination of urine remain the accepted standard method for detecting* S. haematobium i*nfection [9–11]. Recent studies indicate that egg excretion exhibits seasonal variation [12–14] and may have a parallel presentation with seasonal water contact patterns and snail density fluctuations in the transmission rate of* S. haematobium* [15, 16]. In some studies,* S. haematobium* ova excretion in urine have been used as a proxy measure of cercarial infection in intermediate snail host and vector abundance at transmission sites [16–18]. The seasonal transmission of* S. haematobium* varies between countries and even between regions in the same country. In some areas the transmission of* S. haematobium* is higher in the dry season [14, 19, 20], while in other areas, it is higher during the rainy season [21, 22]. Also, perennial transmission has been reported in some areas [21, 23]. Praziquantel has been demonstrated to reduce the level of urinary tract morbidity substantially within six months after treatment [23–25]. Nonetheless the sustainability of morbidity reduction will depend on the level of transmission and thus the level of reinfection after treatment [23]. Augusto et al. [26] confirmed that the transmission season has an influence on parasitological cure rate and intensity of* S. haematobium* infection following PZQ administration.

In Cameroon, urogenital schistosomiasis is mesoendemic in the Bafia Health Area [27–29], South West Region. This health area is made up of three rural communities: Ikata, Bafia, and Munyenge. Studies in these various areas revealed high* S. haematobium* infection rates in school-aged children [30] and pregnant women [29] during the rainy season. Nonetheless, no study has reported the infection level in the dry season. Not only is schistosomiasis influenced by climatic factors, but also human behaviour as well as socioeconomic factors plays a key role in the schistosomiasis transmission process [31–33]. Through various water contacts, the human being ensures the successful transmission of the parasite [34, 35]. Consistent findings showed that the risk of infection among pregnant women in Munyenge is determined by the frequency and intense contact with infested water during bathing and domestic activities [29, 36]. The community-based studies carried out in the study did not assess stream contact behaviour in relation to risk of infection. In endemic areas, it is well established that school-aged children are particularly at risk of infection due to high frequency of contact with infested water [37]. Socioeconomic factors such as educational level and marital status may influence attitudes towards water contact in this area [36, 38]. The first ever community-mass drug administration of PZQ was carried out in the Bafia Health Area in April 2017 with a therapeutic coverage rate of 92.4%. The preventive chemotherapy campaign involved the selective administration of the antischistosomal drug praziquantel to school-aged children [39]. Thus, the present study investigated the prevalence, intensity, and risk factors associated with* S. haematobium* egg excretion in the entire population of the Bafia Health Area during the dry season, six months following the mass distribution of PZQ.

## 2. Methods

### 2.1. Study Area

**Figure 1:**
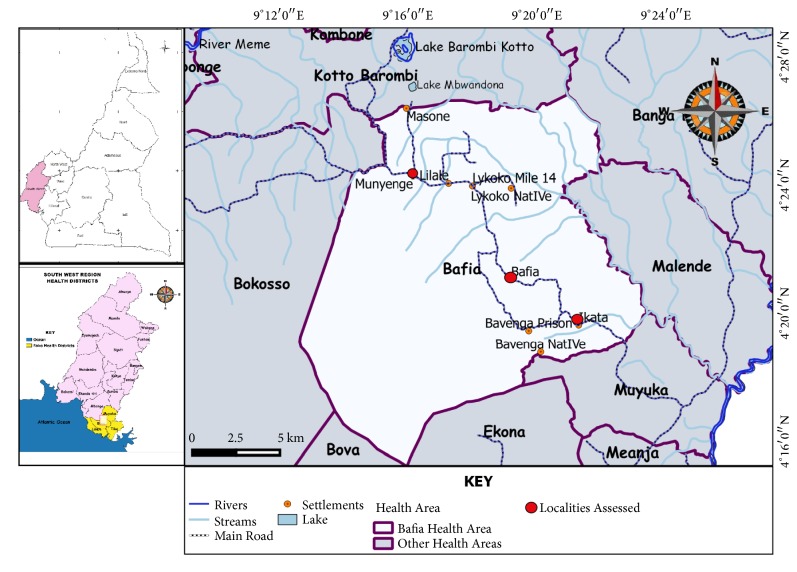
Map showing Bafia Health Area in the Mount Cameroon Area.

This study was carried out in the Bafia Health Area, an endemic focus located in the Mount Cameroon Area. The Bafia Health Area is comprised of three rural communities: Ikata, Bafia, and Munyenge located between longitudes 9.363E and 9.292E and latitudes 4.329N and 4.401N (Figure 1). The altitude of the area ranges from 87 to 168 m above sea level. It has a heterogenous population of about 25,018 inhabitants consisting of individuals from several cultural backgrounds including natives from Oroko, Wimbum, Kom, Metta, Ibo, Ngie, Ndop, and Isimbi [29]. The principal occupation of the people is farming, their main cash crops being cocoa and plantains [30].

The Mount Cameroon Area has an equatorial climate with two distinct seasons: a rainy season which lasts from March to October followed by a short dry season of four months (November to February) [40]. The streams in the Bafia Health Area are suitable habitats for the* Bulinus *species intermediate host and thus constitute the main transmission source of* S. haematobium* infection [36]. The area has a temperature range of about 24-27°C which favours a high release and infectivity of cercariae into the waters [30]. The population uses the streams for drinking water, bathing, washing of clothes, and household utensils. These streams are visited by children as young as 2 years and adults as old as 50 years of age or more [30].

In Ikata and Munyenge, the streams are quite close to houses and schools but in Bafia, the lone spring is far from residential areas [28]. The difference in the number of and proximity to streams may account for the difference in prevalence of UGS reported in these areas [28, 29] (Figure 1). Presently, these communities have pipe-borne sources but not all parts of the communities have access to safe water and sometimes water supply is not regular. Thus, the populations still make frequent use of the streams for their daily needs. Nonetheless, following increased piped water sources in Munyenge,* S. haematobium* infection among pregnant has declined due to reduced stream contact [36].

### 2.2. Study Participants

The study population included individuals of both sexes aged 3-62 years living in the Bafia Health Area. In order to be included in the study, participants must have lived for at least six months in the study area. The sample size of the study population was calculated considering the UGS prevalence of 34.3% in the Bafia Health Area reported by Ebai et al. [28]. The sample size was determined using the Lorenz formula [41]. The total number of samples N is given by N = Z^2^ P (1-P)/d^2^ where Z is the standard normal deviation, Z = 1.96 for the confidence level of 95%, P = 34.3%: proportion of UGS prevalence, and d is the total width of the confidence interval (e.g., 0.05 = ±5). The minimum estimated sample size calculated per locality was 346. For logistics reasons, 349, 334, and 318 consented participants from Munyenge, Ikata, and Bafia were enrolled into the study, respectively. The sample size of each locality is within 90–95% of the expected sample size calculated.

### 2.3. Study Design

The study was a cross-sectional study carried out from November 2017 to January 2018 involving individuals living in the Bafia Health Area. After obtaining administrative and ethical clearances, an acquaintance visit was made to the chiefs and block heads of the localities concerned in order to inform them about the study procedures. A convenient sampling technique was used to recruit participants into the study. Potential participants were informed about the study in their various localities and only those who met the inclusion criteria and voluntarily gave their consent/assent were enrolled. For children less than 13 years old, assent was gotten from their parents or legal guardians, while for those less than 18 years old, consent was obtained from both the parent and the child. Participants who were 18 years and above gave their consent. The participants were requested to rally at the residence of their respective block heads on the day programmed for sample collection. A semistructured questionnaire was administered to the participants to obtain data on sociodemographic factors and stream contact behaviour. Urine samples were collected and processed for the detection of microhaematuria and* S. haematobium *ova.

#### 2.3.1. Administration of Questionnaire

Participants were interviewed by a field researcher using a semistructured questionnaire to obtain information on demographic (age, sex, and residence), socioeconomic indicators (educational level and occupation), and stream contact behaviour (household water source, number of streams, stream usage, frequency of contact with open water source, and activities carried out in the stream).

#### 2.3.2. Sample Collection and Laboratory Analysis

Each participant who filled the questionnaire was given a sterile, wide mouthed, screw capped plastic bottle carrying their identification number, age, and gender and was properly instructed on how to collect the urine sample between 10 am and 2 pm. The younger children (aged ≤ 12 years) collected their urine with the help of their parents. Immediately after collection, samples were tested for microhaematuria using urine reagent strips (Uripath, Plasmatec Laboratory, UK) (Combi-11) as per manufacturer instructions. Results were expressed as negative or in levels of positivity (+, ++, or +++) excluding presence of traces. All samples were transported on ice bath to the University of Buea, Medical Research Laboratory, and processed within 24 hours of collection.

*Schistosoma haematobium *ova were identified in urine samples using the filtration technique as described elsewhere [42]. In brief, 10 mL of urine was filtered using membrane filters (Sterlitech Polycarbonate (PCTE) membrane filters, USA) and the egg count was recorded per 10mls of urine. The infection intensity was classified as light (<50 eggs/10 mL of urine) or heavy (≥50 eggs/10 mL of urine) as defined by the World Health Organization (WHO) [43]. Microhaematuria was considered as proxy-diagnosis of UGS, an accepted marker in the rapid diagnosis of* S. haematobium* infection in urine [44]. Thus, an individual was positive for* S. haematobium *when he/she was diagnosed positive by microscopic examination and/or urine reagent strip.

### 2.4. Ethical Considerations

The administrative and ethical clearances to conduct the study were obtained from the Regional Delegation of Public Health, South West Region, and Faculty of Health Sciences Institutional Review Board (Ref: 2017/098/UB/SG/IRB/FHS), respectively. Meetings were held with the local authorities of the area to inform them about the study aims, design, benefits, and risks involved and thereafter possible dates and venues scheduled for collection of data and sample. Community health workers were used to inform the communities on prospective study and dates selected for the study. Signed assent and/or consent form indicated voluntary participation. All participants who were positive for UGS were freely treated with praziquantel tablets (40 mg/kg of body weight) in collaboration with the local health authorities. Each participant was free to withdraw consent at any time. Identification codes were assigned to each study participant and limited access to study data was maintained to ensure confidentiality.

### 2.5. Statistical Analysis

The data were analyzed using SPSS version 21.0 (SPSS, Inc., Chicago, IL, USA). Proportions of* S. haematobium *infection were compared between different groups (age groups, sex, area of residence, educational level, occupational status, household water source, stream usage, and frequency to stream) using Pearson Chi-square test. The Kruskal-Wallis test (H) was used to compare the differences in intensities of infections. Odd ratios (OR) and confidence intervals (CIs) were calculated using a Microsoft Excel confidence interval calculator as described by Armitage & Berry [45] and Newcombe [46]. Variables that had a p-value < 0.20 in bivariate analysis or explanatory plausibility were included in the multivariate logistic regression model for analysis of risk factors for* S. haematobium* egg excretion. A p-value of < 0.05 was considered significant.

## 3. Results

### 3.1. Characteristics of the Study Population

A total of 1001 participants from the three localities were enrolled into the study (Munyenge: 349, Ikata: 334, Bafia: 318), more of them being females. The age of the study participants ranged from 3 to 62 years and only about a third of them had obtained some form of secondary education. A greater percentage of the participants were pupils and farming is a common practice in these communities. Almost all (97.6%) participants reported stream usage for household chores and/or bathing. Despite presence of some piped water sources in this area, stream usage is predominant; meanwhile the coverage rate for piped water usage as the only source of water is low. The characteristics of the study population are shown in Table 1.

### 3.2. Prevalence and Intensity of* S. haematobium* Egg Excretion

**Figure 2:**
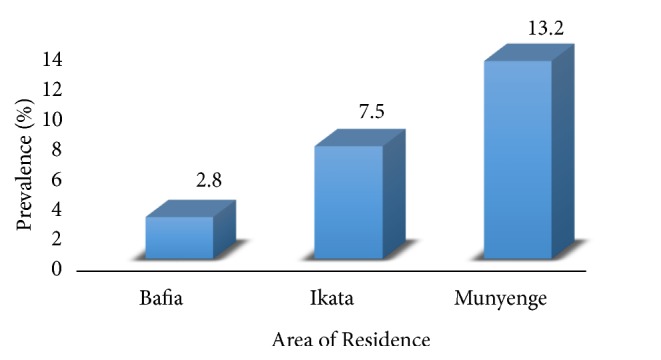
Prevalence of* S. haematobium* egg excretion in the three localities in the Bafia Health Area.

Eighty (8%; 95% CI: 6-10) of the 1001 participants enrolled were positive for* S. haematobium* infection with varying rates in egg excretion among the three localities (Munyenge (13.2%), Ikata (7.5%), and Bafia (2.8%)) as shown in Figure 2. The difference in prevalence of egg excretion was statistically significant (*χ*2 = 24.42, p < 0.001). In general, the intensity of egg excretion was low given an overall mean egg load of 33.5 eggs/10 ml of urine (range: 2-200). Although not statistically significant (Kruskal-Wallis test, H = 2.01, df = 2, p = 0.37), the mean egg load was high in Munyenge (36.36 range: 2-200) (n = 33) when compared with Ikata (16.25 range: 2–57 (n = 4) and Bafia (8.0) (n = 1).

### 3.3. Risk Factors Associated with* S. haematobium* Egg Excretion during the Dry Season in the Bafia Health Area

In bivariate analysis, there was an association between* S. haematobium* egg excretion, age (p = 0.03), locality (p < 0.001), and frequency of contact with stream (p < 0.001) (Table 2). Surprisingly, children in age group < 5 years had more infection (2.54; 95% CI: 1.04–4.79) when compared with the older age groups. However, the difference was not significant. All individuals who had piped water as the only source of water were diagnosed negative for the infection (Table 2). No association was seen between gender, level of education, occupation, source of water, stream activity, and infection status (Table 2). All three factors associated with infection were retained by multiple regression model analysis (Table 2). Gender and educational level have been identified as plausible explanatory variables to the prevalence of schistosomiasis and thus were included in the final model.

The risk of egg excretion increased by 4.79 times (95% CI: 2.20-10.41) and 3.68 times (95% CI: 1.59-8.54) among residents in Munyenge and Ikata, respectively. It worth noting that there was a statistical difference (*χ*^2^ = 58.73; p < 0.001) between locality and frequency to the stream where people living in Munyenge (20%; 70/139) frequent (> thrice/day) the stream more than those from Ikata (11.9%; 37/139) and Bafia (10.1%; 32/139). More so, frequent contact with stream correlated with higher odds of egg excretion where more than three visits/days to the stream were associated with highest odds of egg excretion (OR: 8.43 95% CI: 3.71-19.13) (Table 2). The age group (5–15 years) was less likely (OR: 0.42 95% CI: 0.19-0.91) associated with* S. haematobium *egg excretion.

### 3.4. Association between Age, Sex, and Stream Contact Behaviour

We assessed the level of exposure to infested water among the different age and sex groups. The age group (5 -15 years) was significantly associated with more exposure to infested streams. Stream usage (*χ*^2^ = 19.97; p < 0.001), intense (both domestic chores and bathing) (*χ*^2^ = 48.98; p < 0.001), and frequent (> thrice/week) contact (*χ*^2^ = 202.88; p < 0.001) with water was higher among children between 5 and 15 years than any other age group (Table 3). Bathing activity only was observed more among the age group < 5 years; meanwhile the older age group (> 15 years) visited the stream frequently for domestic chores only. Type of stream activity was gender-related where bathing was more associated with males whereas domestic chores were carried out more by females. The difference was significant (*χ*^2^ = 20.84; p < 0.001) (Table 3).

## 4. Discussion

Urogenital schistosomiasis is meso-hyperendemic in the Bafia Health Area and thus a major public health concern in the South West Region of Cameroon. As the streams are the only source of permanent water supply for the community, it is exceedingly difficult to prevent the community from contacting this essential source of water on a daily basis for various uses (bathing; other domestic uses) [27–30]. Given that the first ever community-mass drug administration of PZQ was carried out in the Bafia Health Area in April 2017 to treat and prevent* S. haematobium* infection among school-aged children, this study reports on the prevalence, intensity, and risk factors associated with* S. haematobium* egg excretion in the area during the dry season, six months following the mass drug campaign.

In the Bafia Health Area, the prevalence of* S. haematobium* egg excretion during the dry season was 8%. This prevalence is considerably low than the levels reported in the same area during the rainy season by previous authors. Ntonifor et al. [30] reported a prevalence of 40.27% in Munyenge, while a prevalence of 34.3% was reported by Ebai* et al. *[28] in the Ikata-Likoko area. The low prevalence of* S. haematobium* in the present study is most likely attributed to the preventive chemotherapy campaign carried out in the Bafia Health Area. Drug-enhanced protective immune mechanisms against the infection may account for a greater reduction in egg output observed during the dry season. It is well established that PZQ kills mainly the mature worms [47, 48] and is less effective against juvenile (2- to 4-week-old) parasites [48, 49]. The cumulative deaths of adult* S. haematobium* worms provide the main source of protective antigen that boost antibody responses associated with protection against reinfection [50] as well as reduction in* S. haematobium* fecundity [51]. However, the presence of infection after PZQ treatment may be due to infection just prior to treatment, reinfection after treatment, or treatment failure. In high transmission areas, the removal of adult worms by treatment may result in low cure rates due to the development of immature worms into egg-producing adults by the time of the follow-up assessment of cure [48]. The low prevalence of egg excretion recorded in the human host during the dry season may as well parallel low transmission potentials at the water contact sites during this period. Ivoke et al. [18] suggested that the community urinary egg output may be a determinant of the infection rate of the snail intermediate host as well as the dynamics of intramolluscal larval population at the transmission sites. Nevertheless, the annual transmission pattern for UGS in Bafia Health Area remains to be determined and malacological surveys are imperative in investigating the dynamics of snail host infectivity and cercarial output in this setting.

Residents from Munyenge and Ikata localities were almost five and four times, respectively, more likely at risk of* S. haematobium* egg excretion than those living in Bafia. In Munyenge, individuals had the tendency to make frequent visits to the stream than those from Ikata and Bafia communities given the presence of more streams and closeness of houses to the streams in this rural community. This results support the well documented observation that people living near the transmission site of schistosomiasis are usually more infected [34, 35]. Similarly, Ebai et al. [28] reported geographical risk associated with infection in the health area. Although not significant, a high intensity of egg excretion was recorded in Munyenge compared with Ikata and Bafia. This may reflect differing transmission intensities among the areas. There is an inverse relationship between PZQ cure rate and intensity of infection in schistosomiasis [52, 53]. It is plausible that children from Bafia had a better cure rate compared with those from more exposed communities.

The prevalence of* S. haematobium* infection recorded among school children contrasts other studies in Cameroon [30, 54] as well as other parts of Africa [55], which reported peak prevalence of UGS among school-aged children. In corroboration with the findings of Houmsou [21], in Nigeria, children of preschool age were found to have a higher prevalence of egg excretion (OR = 2.54) than those of the older age groups. The considerable prevalence in this group could be the result of early exposure to infested water bodies when these children are taken along with their mothers or caregivers [21] to water sources for domestic chores, where they often play and swim naked in the infested water [56, 57]. Similarly, bathing/swimming activity was reported more among the youngest age group. Preschool-aged children are usually susceptible to infections due to a weaker immune system [58]. Several studies of UGS tend to focus on school-aged children and adults, with little or no emphasis on preschool children. These neglected groups (pregnant women inclusive) which are believed not to be sufficiently exposed to infection and often left untreated could serve as reservoirs of infection, bringing the distribution of the disease to precontrol level over time [59]. Besides, untreated infected children may develop severe consequences such as malnutrition, reduced ability and impairment of cognitive development which may jeopardise their well-being later in life [4]. In endemic areas, school-aged children are particularly at risk of infection due to high frequency of contact with infested water [37]. Contrary to other studies, our findings revealed that this age group (5-15 years) was less likely associated with egg excretion despite frequent and intense contact with stream. The selective mass distribution of praziquantel in this area involved school-aged children and may explain the decreased infection prevalence observed among this age group. Targeted control of schistosomiasis on school children is often advocated and is usually the main operation in sub-Saharan Africa [59]. Treatment of schistosome infections with praziquantel (PZQ) enhances schistosome-specific immune responses which are associated with protection against reinfection [50] as well as reduction in* S. haematobium* fecundity [51]. In line with earlier studies carried out in South West Region [29, 30, 60], infection status was comparable between the different sexes although water contact activities were sex-related in this endemic setting. Bathing and domestic chores were associated with the male and female gender, respectively. Studies have reported sex dependent pattern of infection [18]. The lack of association between infection and sex may be due to the fact that both activities equally cause the most exposure to cercaria-infested water [61].

It has been observed that water contact patterns are often the major factors with regard to the spatial distribution of* S. haematobium* infection. Consistent with previous reports in Bafia Health Area, frequent and intense contact with stream were the main factors associated with increased risk of infection [29, 36]. In this study, community water contact at any point in time is mostly linked to practices including domestic activities and bathing. Leisure water contact particularly during washing of clothes and bathing was the major types of water contact activities significantly associated with school-aged children (5-15 years) in the study area. Laundry, bathing, and recreational swimming are the activities that cause the most exposure to cercaria-infested water because these do involve the immersion of large body parts, for long periods [61]. Recent findings strongly suggest that the extension of more piped water sources in this endemic area will reduce the incidence of infection by reducing the need for intense or frequent contact with infested water [36].

## 5. Conclusions

The prevalence of* S. haematobium* egg excretion is low in the Bafia Health Area during dry season and most likely attributed to the preventive mass chemotherapy campaign with PZQ conducted in the area, six months prior to the study. The low prevalence of egg excretion in the human host may as well parallel low transmission potentials at the water contact sites during this period. The low ova excretion associated with school-aged (5–15 years) children is a likely evidence of the effectiveness of the mass treatment campaign. Since the risk of infection in this area is determined mainly by water contact behaviour, changing the frequency and/or nature of water contact in these communities either through provision of adequate supply of portable water or through health education is a feasible means of preventing and interrupting UGS transmission in the Bafia Health Area.

## Figures and Tables

**Table 1:** Sociodemographic characteristics of the study participants.

Variable	Characteristic	Number examined (n)	Percentage (%)
Area of residence	Munyenge	349	34.9
	Ikata	334	33.4
	Bafia	318	31.8

Sex	Female	568	56.7
	Male	433	43.3

Age group (years)	< 5	86	8.6
	5 - 15	476	47.6
	16 - 39	270	27.0
	≥ 40	169	16.9

Educational level	Primary	671	67.0
	Secondary	330	33.0

Occupation	Farmer	356	35.6
	Pupil	436	43.6
	Student	185	18.5
	Housewife	24	2.4

Stream usage	Yes	977	97.6
	No	24	2.4

Source of water	Stream only	637	63.6
	Stream and piped borne water	340	34.0
	Piped only	24	2.4

Stream activity	Bathing only	126	12.9
	House chores only	362	37.05
	House chores and bathing	489	50.05

Frequency to stream/daily	Once	303	31.01
	Twice	272	27.84
	Thrice	192	19.65
	>Thrice	139	14.23
	Hardly	71	7.27

**Table 2:** Risk factors associated with *S. haematobium* egg excretion during the dry season.

Variable	Category	*S. haematobium*	Unadjusted OR	^#^Adjusted OR	P- value
		Positive % (n)	(95% CI)	(95% CI)	
Area of residence	Munyenge	13.2 (46)	5.21 (2.51-10.83)	4.79 (2.20-10.4)	<0.001
	Ikata	7.5 (25)	2.78 (1.27-6.05)	3.68 (1.59-8.54)	0.002
	Bafia	2.8 (9)	REF	REF	
	*χ* ^2^; P value	24.416; <0.001			

Sex	Male	8.8 (38)	1.21 (0.76-1.91)	NA	
	Female	7.4 (42)	REF		
	*χ* ^2^; P value	0.638; 0.425			

Age group(years)	< 5	16.3 (14)	2.54 (1.12-5.77)	2.0 (0.81-4.94)	0.13
	5 - 15	6.9 (33)	0.97 (0.49-1.93)	0.42 (0.19-0.91)	0.028
	16 - 39	7.8 (21)	1.20 (0.58-2.52)	0.71 (0.32-1.60)	0.41
	≥ 40	7.1 (12)	REF	REF	
	*χ* ^2^; P value	8.958; 0.030			

Educational level	Primary	9.1 (61)	1.64 (0.96-2.79)	1.59 (0.88-2.85)	0.124
	Secondary	5.8 (19)	REF	REF	
	*χ* ^2^; P value	3.492; 0.322			

Occupation	Farming	7.9 (28)	1.23 (0.61-2.48)	NA	
	Pupil	9.2 (40)	1.46 (0.74-2.84)		
	Student	6.5 (12)	REF		
	Housewife	0 (0)	----------		
	*χ* ^2^; P value	5.48; 0.360			

Source of water	Stream only	8.2 (52)	0.99 (0.61-1.60)	1.1 (0.30-3.72)	0.92
	Stream and piped water	8.2 (28)	REF	REF	
	Piped water only	0 (0)	----------	----------	------
	*χ* ^2^; P value	2.137; 0.343			

Stream activity	House chores and bathing	8.0 (39)	1.28 (0.58-2.81)	0.62 (0.27-1.47)	0.28
	House chores only	9.1 (33)	1.48 (0.66-3.29)	1.03 (0.42-2.51)	0.95
	Bathing only	6.3 (8)	REF	REF	
	*χ* ^2^; P value	1.011; 0.603			

Frequency to stream++ per day	More than thrice	18.0 (25)	5.82 (2.77-12.21)	8.43 (3.71-19.13)	<0.001
	Thrice	9.9 (19)	2.91 (1.35-6.27)	4.48 (1.93-10.41)	<0.001
	Twice	7.7 (21)	2.22 (1.05-4.69)	3.63 (1.64-8.01)	0.001
	Hardly	5.6 (4)	1.58 (0.49-5.13)	1.07 (0.31-3.72)	0.92
	Once	3.6 (11)	REF	REF	
	*χ* ^2^; P value	27.23; <0.001			

*χ*2 = Pearson Chi-square test; OR = odd ratio; aOR = adjusted OR using multivariate regression analysis.

**Table 3:** Association between age, gender, and stream contact behavior.

Stream contact behaviour	Age group (years) % (n)				Gender % (n)	
*Stream usage*	<5	5 - 15	16 - 39	≥ 40	Female	Male

Yes	93.0(80)	99.6(474)	97.0(262)	95.3(161)	98.1(558)	97.0(419)

No	7.0(6)	0.4(2)	3.0(8)	4.7(8)	1.9 (11)	3.0(13)

*χ*^2^; P value	19.970; <0.001				1.215; 0.301	

*Activity at stream*						

Bathing	21.3(17)	15.6(74)	7.3(19)	9.9(16)	10.4(58)	16.2(68)

House chores	37.5(30)	27.6(131)	45.4(119)	50.9(82)	42.8(239)	29.4(123)

Both	41.3(33)	56.8(269)	47.3(124)	39.1(63)	46.8(261)	54.4(228)

*χ*^2^; P value	48.984; <0.001				20.838; <0.001	

*Frequency to stream per day*						

Hardly	22.5 (18)	1.7(8)	3.1(8)	23.0(37)	8.1(45)	6.2(26)

Once	47.5(38)	20.7(98)	37.0(97)	43.5(70)	30.6(171)	31.5(132)

Twice	17.5(14)	31.9(151)	29.0(76)	19.3(31)	27.6(154)	28.2(118)

thrice	3.8(3)	26.6(126)	17.9(47)	9.9(16)	18.6(104)	21.0(88)

More than thrice	8.8(7)	19.2(91)	13.0(34)	4.3(7)	15.1(84)	13.1(55)

*χ*^2^; P value	202.884; <0.001				2.528; 0.640	

*χ* ^2^ = Pearson Chi-square test.

## Data Availability

All datasets on which the conclusions of the research rely are presented in the paper. However, data is available from the corresponding author upon reasonable request.

## Abbreviations

UGS: Urogenital schistosomiasis
OR: Odd ratio
CI: Confidence interval
SWR: Southwest region
PZQ: Praziquantel
H: Kruskal-Wallis test
*χ*^2^: Pearson Chi-square test
^#^OR: Adjusted odd ratio.
